# MicroRNA-378 Regulates Adiponectin Expression in Adipose Tissue: A New Plausible Mechanism

**DOI:** 10.1371/journal.pone.0111537

**Published:** 2014-11-07

**Authors:** Masayoshi Ishida, Michio Shimabukuro, Shusuke Yagi, Sachiko Nishimoto, Chisayo Kozuka, Daiju Fukuda, Takeshi Soeki, Hiroaki Masuzaki, Masato Tsutsui, Masataka Sata

**Affiliations:** 1 Department of Cardiovascular Medicine, The University of Tokushima Graduate School of Health Biosciences, Tokushima, Japan; 2 Department of Cardio-Diabetes Medicine, The University of Tokushima Graduate School of Health Biosciences, Tokushima, Japan; 3 Department of Nutrition and Metabolism, The University of Tokushima Graduate School of Health Biosciences, Tokushima, Japan; 4 Department of Pharmacology, Graduate School of Medicine, University of the Ryukyus, Okinawa, Japan; 5 Division of Endocrinology, Diabetes and Metabolism, Hematology and Rheumatology (Second Department of Internal Medicine), Graduate School of Medicine, University of the Ryukyus, Okinawa, Japan; The University of Tokyo, Japan

## Abstract

**Aims:**

Mechanisms regulating adiponectin expression have not been fully clarified. MicroRNAs (miRNAs), small non-coding RNAs that regulate gene expression, are involved in biological processes, including obesity and insulin resistance. We evaluated whether the miRNA-378 pathway is involved in regulating adiponectin expression.

**Methods and Results:**

First, we determined a putative target site for miRNA-378 in the 3 prime untranslated region (3'UTR) of the adiponectin gene by *in silico* analysis. The levels of adiponectin mRNA and protein were decreased in 3T3-L1 cells overexpressing the mimic of miRNA-378. Luminescence activity in HEK293T cells expressing a renilla-luciferase-adiponectin-3'UTR sequence was inhibited by overexpressing the mimic of miRNA-378, and the decrease was reversed by adding the inhibitor of miRNA-378. Moreover, we confirmed the inhibitory effects of the mimic were cancelled in a deleted mutant of the miR-378 3′-UTR binding site. Addition of tumor necrosis factor-α (TNF**α**) led a upregulation of miR-378 and downregulation of adiponectin at mRNA and protein levels in 3T3-L1 cells. Level of miR-378 was higher and mRNA level of adiponectin was lower in diabetic ob/ob mice than those of normal C57BL/6 mice and levels of miR378 and adiponectin were negatively well correlated (r = −0.624, p = 0.004).

**Conclusions:**

We found that levels of miRNA-378 could modulate adiponectin expression via the 3'UTR sequence-binding site. Our findings warrant further investigations into the role of miRNAs in regulating the adiponectin expression.

## Introduction

Adipose tissue secretes adipocytokine/adipokine, which play critical roles in energy and vascular homeostasis [1], [2]. Regulation of these adipocytokine/adipokine triggers the development of a pro-inflammatory state, which is considered to form the “common soil” for the pathogenesis of obesity-linked disorders [1], [2]. Adiponectin, an anti-inflammatory and insulin-sensitizing molecule, has emerged as a master regulator of inflammation/immunity in various tissues, including adipose tissue, its own assembly site [3], [4]. Expression levels of adiponectin are stimulated by insulin-sensitizing thiazolidinediones, indicating that the adiponectin gene is a target of PPARγ (peroxisome-proliferator activated receptor γ) [5]. Moreover, the expression of the adiponectin gene is known to be regulated positively by C/EBP (CCAAT/enhancer-binding protein) α, SREBP (sterol-regulatory-element-binding protein)-1c, FoxO1 (forkhead box O1) and Sp1 (specificity protein 1), and negatively by pro-inflammatory factors, such as reactive oxygen species (ROS), TNFα (tumor necrosis factor-α) and IL (interleukin)-6 [6]. It is known that adiponectin gene expression in adipose tissues is down-regulated in subjects with obesity and insulin resistance [3], [4]; however, the mechanisms of the down-regulation are largely unknown. Since hypoadiponectinemia has links to metabolic and cardiovascular abnormalities associated with obesity and insulin resistance [7], [8], exploring the mechanisms regulating adiponectin expression and post-translational modification is crucial for understanding obesity-linked disorders.

MicroRNAs (miRNAs) are short (19–22 nucleotides), evolutionarily conserved, non-coding RNA molecules involved in gene regulatory functions. They operate through a mechanism involving complementary sequence binding to the 3'UTR region of a target mRNA molecule. Formation of a miRNA: mRNA complex results in either increased degradation of the target mRNA molecule [9], or inhibition of target mRNA translation [10]. miRNA-mediated regulation has also been observed in adipose tissue [11]–[13]. In subsets of miRNAs that declined, increased, or remained unchanged during conversion, Gerin et al. discovered a highly induced locus within the intron of proliferator-activated receptor γ coactivator 1β (PGC-1β) that encodes miRNAs 378 [13]. They subsequently showed that overexpression of miRNA-378 upregulated a set of lipogenic genes, suggesting the involvement of PPARγ_2_ machinery [13].

From the above, we hypothesized that the miRNA pathway could be involved in regulation of adiponectin expression. By searching *in silico*, we found a putative target site for miRNA-378 in the 3 prime untranslated region (3'UTR) of the adiponectin gene (*Adipoq*). To clarify the roles of miRNA-378 in adiponectin gene expression, we evaluated variation in adiponectin, miRNA-378, and its related molecules, PPARγ_2_ [13], [14], PGC-1β [13], [15], and estrogen-receptor-related receptor γ (ESRRG) [16] in white adipose tissue; second, we quantitated adiponectin level during adipogenesis in 3T3-L1 cells overexpressing the mimic or inhibitor of miRNA-378; finally, we assessed regulation of adiponectin expression by miRNA-378 in HEK293T cells expressing a luciferase-adiponectin-3'UTR sequence or a mutated construct.

## Materials and Methods

### *In silico* prediction of miRNA-378 targets

To predict miRNA-378 targets, we used TargetScan (www.targetscan.org) and PicTar (www.pictar.org).

### Cell lines and induction of mature adipocyte

The mouse 3T3-L1 cell line was maintained in DMEM, 10% FBS (Sigma), 1% penicillin/streptomycin (Sigma). The cells were dispensed into 6-well plates before induction of adipose differentiation. Once the cells had grown confulently, they were stimulated with insulin (10 µg/mL, Sigma), dexamethasone (1 nM, Sigma), and IBMX (500 µM, Sigma) for 2 days, and medium was changed to complete DMEM every 2 days. Overexpression and knockdown of miRNA-378 were done by transfecting miScript miRNA-378 mimic (pre-mir) and/or inhibitor (anti-sense) (QIAGEN) with RNAiMax transfection reagent (Life Technologies) on day 4 according to the manufacturer's instruction. To explore the function of miR378 on the pathological conditions, we stimulated 3T3-L1 adipocyte with human recombinant TNFα (10µg/mL, R&D systems) on day 6 and levels of adiponectin expression were evaluated by quantitative reverse transcription PCR (qRT-PCR) or Western blotting.

### Animals

From normal C57BL/6 (♂, n = 10, body weight 30.4±1.5 g) or diabetic ob/ob mice (♂, n = 10, body weight 47.7±1.6 g, casual blood glucose level 178±30 mg/dL), epididymal adipose tissues were excised and used for gene expression analyses. Animal experiments were approved by the Committee on Animal Research, the University of Tokushima and have been conducted in accordance with international ethical principles and guidelines for experiments on animals.

### Gene expression analyses

Total RNAs were purified from mouse adipose tissue samples using RNeasy Lipid Tissue Mini kit (QIAGEN KK, Tokyo), and total RNAs from cultured cells were purified with the Total RNA Purification Kit (Norgen Biotek, Canada). To detect miRNA-378 and U6 RNA polymerase (as an internal control) expression levels, we used the TaqMan MicroRNA Reverse Transcription Kit and TaqMan primer/probe sets for miRNA-378 and U6 (Applied Biosystems), respectively, as per their protocols. Total RNAs were reversed-transcribed using the QuantiTect RT kit (QIAGEN), and then qRT-PCR was done using the Power SYBR Green Kit (Applied Biosystems) with an Mx3000 thermocycler (Stratagene). Specific primers for the genes of interest are shown in **Table S1**. All data were analyzed by the ΔΔCt method, using β-actin (for genes) or U6 (for miR-378) as internal controls, respectively.

### Western blotting

The 3T3-L1 cells were lysed in RIPA buffer (Wako Pure Chemical Industries, Japan) supplemented with protease inhibitor cocktail (Roche) for 30 min on ice and centrifiguted at 10^4^ x *g* for 10 min to obtain cell lysates. The resultant lysate was run on 4–12% Tris-Glycine NuPAGE gels (Life Technologies) and transferred onto PVDF membrane (Bio-Rad). Membranes were blocked with 5% skimmed milk in PBS-T at room temperature for 2 h and incubated with rabbit anti-adiponectin (#2789S, Cell Signaling Technologies) or mouse anti-β-actin monoclonal (Clone AC-15, Sigma) at 4°C overnight. Membranes were washed 3X with PBS-T and incubated with HRP-conjugated anti-rabbit IgG (#7074, Cell Signaling Technologies) or HRP-conjugated anti-mouse IgG (BD Biosciences) in 5% skimmed milk in PBS-T at room temperature for 1hr. After washes 5 X with PBS-T, the bands were visualized by enhanced chemiluminescence (ECL, Perkin-Elmer) and quantified using by a CCD camera system (LAS4000mini, GE Healthcare) with ImageQuant TL software (GE Healthcare).

### Cloning of the 3′UTR of mouse *Adipoq*

The mouse adiponectin gene *(Adipoq)* 3′-untranslated region (3′-UTR) (+859–1211; total 352 bp) was amplified from 3T3-L1-derived adipocyte cDNA by PCR using the high fidelity Phusion polymerase (Thermo Fisher Scientific Inc.) and primers ATATCTCGAGCTGCAACTACCCATAGCCCA (forward) and ATATGCGGCCGCGTGAGAAGAGTAATCACTGT (reverse). The PCR product was then cloned between the XhoI and NotI sites downstream of the Renilla luciferase gene in the psiCheck2 vector (Promega), and the resultant construct was confirmed by DNA sequencing.

### Renilla-luciferase activity

HEK293T cells were dispensed into 24-well or 96-well plates and co-transfected with psiCheck2 (Promega) carrying the *Adipoq* 3′UTR or ΔmiR-378 binding site (BS) (0.5 µg/well in a 24-well plate or 0.2 µg/well in a 96-well plate) and mimic or inhibitor of miRNA-378 using RNAiMax (Life Technologies) as per the manufacturer's protocol. After 48 h, cells were lysed in 1× Passive Lysis buffer provided with the Dual-Luciferase Reporter Assay System (Promega, USA). At least 3 independent transfections were done in triplicate, and luciferase activities were measured with a luminometer.

### Site-directed mutagenesis

The above vector construct was further mutagenized to introduce point mutations to delete miR-378 binding sites with High Fidelity Phusion polymerase and primers, AAATAATTTGTGTTCCTAgaattcAAAAAAAGGCACTCCC (Forward, *EcoRI* site in underlined) and GGGAGTGCCTTTTTTT gaattc TAGGAACACAAATTATTT (reverse). The reaction was then digested with 5 units of Dpn I (Thermo Scientific) to eliminate the parental vector at 37°C for 2hr. The Dpn I-treated PCR product was then transformed to DH5α competent cells (TaKaRa Bio) to obtain the mutagenized clones. The clone obtained was confirmed by DNA sequencing.

### Statistical analysis

Values are expressed as mean (SE). Levels of qPCR measurements are expressed as logarithm to the base 10 otherwise indicated. Unpaired *t*-test or one-way ANOVA (for parametric group comparison) and Kruskal-Wallis or Wilcoxon-Mann-Whitney tests (for non-parametric comparison) were employed. Pearson product-moment correlation coefficients (r) were determined to measure the strength and direction of the linear relationship between levels of adiponectin, miR378 and other variables. All analyses were performed using Jump version 11.2.0 software (SAS Institute Inc., Cary, NC). Probability was considered significant if p<0.05.

## Results

### miRNA-378 in 3T3-L1 adipocytes

**Figure 1:**
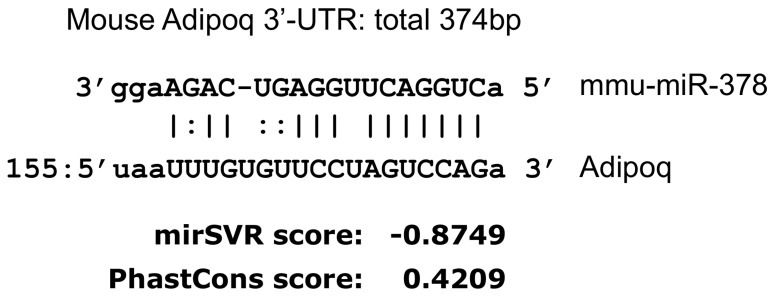
Prediction of miRNA-378 targets *in silico*. The miRNA-378 targets were predicted using Targetscan (www.targetscan.org) and PicTar (www.pictar.org).

**Figure 2:**
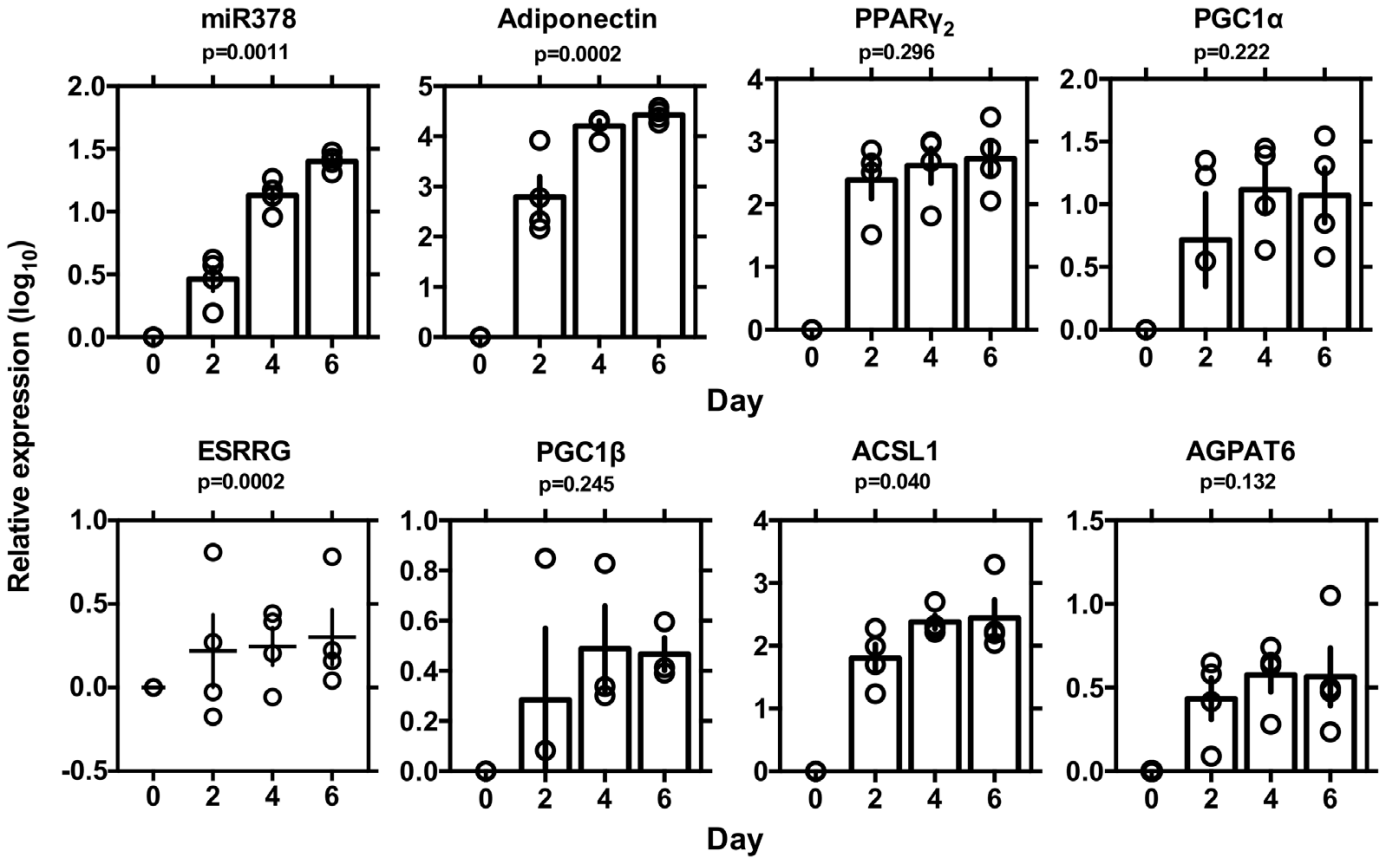
Levels of miRNA-378, adiponectin, PPARγ_2_, PGC1α, ESRRG, PGC1β, ACSL1 and AGPAT6 during differentiation of 3T3-L1 adipocyte cells. The mouse 3T3-L1 cell line was cultured in DMEM/10% FBS and dispensed into 12-well plates (day 2) before induction of adipose differentiation. Once the cells had grown confluently, they were stimulated with insulin (10 µg/mL) dexamethasone (1 nM), and IBMX (500 µM) for 2 days (days 0–2), and the medium was changed every 2 days. At days 0, 2, and 6, RNA was extracted and levels of miRNA-378 and mRNAs of adiponectin, ACSL1, GPAT6, PPARγ2, PGC1α, PGC1β, and ESRRG were evaluated by qPCR. Expression levels normalized to those of β-actin were log-transformed. Individual values are shown with mean ± SE (n = 4).

Searching *in silico*, we found a putative target site for miRNA-378 in the 3'UTR of the adiponectin gene (**Figure 1**). To investigate the effects of miRNA-378 on the expression levels of adiponectin, we performed *in vitro* experiments, beginning by analyzing the abundance of miRNA-378 and adiponectin-related molecules during preadipocyte differentiation in 3T3-L1 cells, using qRT-PCR (**Figure 2**). During differentiation, expression of miRNA-378 and adiponectin were increased. mRNA levels for the lipogenic enzymes ACSL1 (acyl-CoA synthetase long-chain family member 1) and AGPAT6 (1-acylglycerol-3-phosphate acyltransferase 6) increased coordinately with those of the transcriptional factor, PPARγ_2_, and its coactivator, PGC-1α. Simultaneously, mRNA for PGC-1β, which contains an RNA hairpin that generates miRNA-378, and that for ESRRG, which is one of the nuclear receptors coactivated by PGC-1β and is targeted by miRNA-378 at two regions within the 3'UTR, increased during preadipocyte differentiation.

### Overexpressing the mimic or inhibitor of miRNA-378

**Figure 3:**
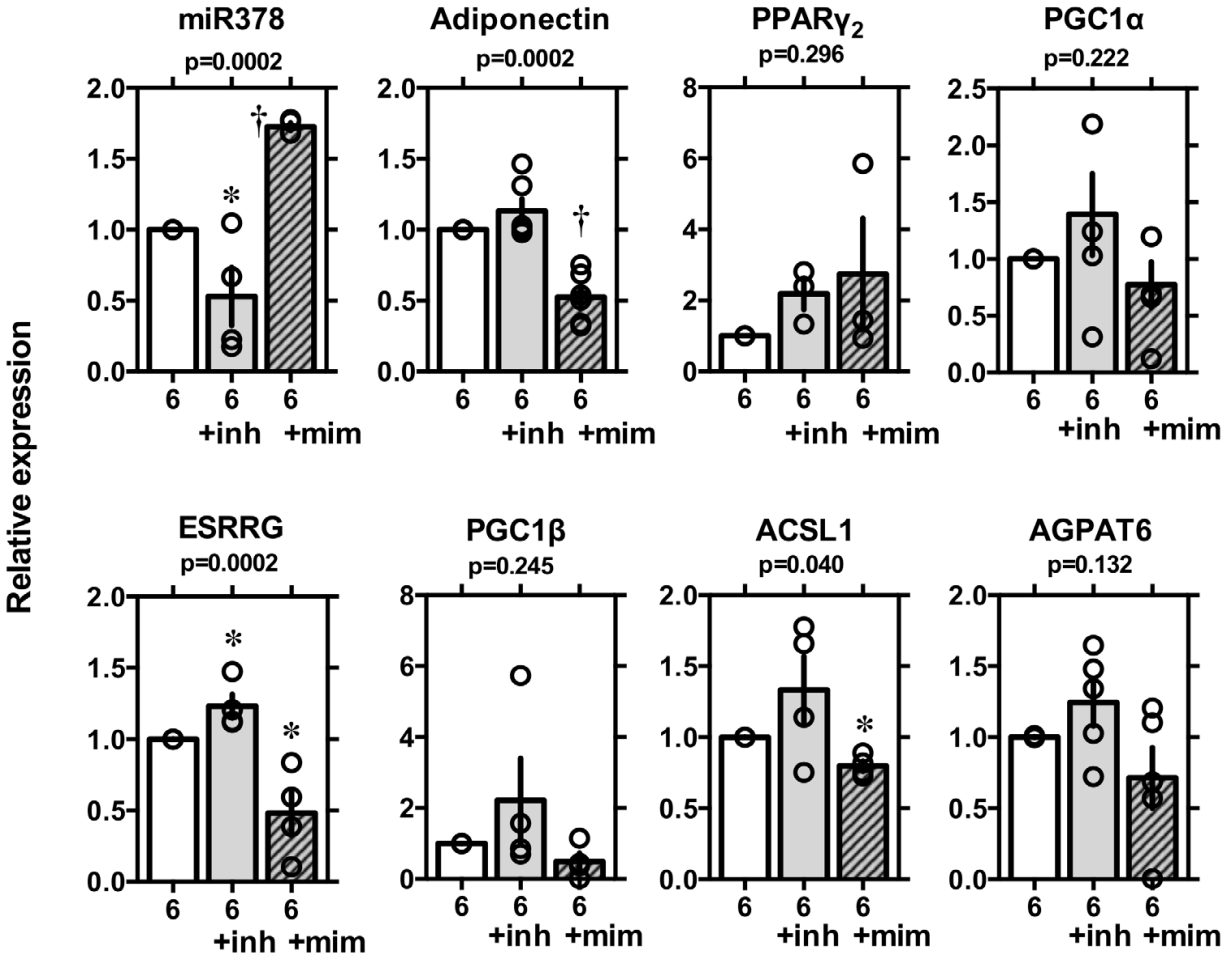
Levels of miRNA-378, adiponectin, PPARγ_2_, PGC1α, ESRRG, PGC1β, ACSL1 and AGPAT6 in 3T3-L1 adipocytes overexpressing the inhibitor (inh) or mimic (mim) of miRNA-378. The mouse 3T3-L1 cell line was cultured in DMEM/10%FBS and dispensed into 12-well plates (day 2) before induction of adipose differentiation. Once the cells had grown confluently, they were stimulated with 1.7 mM insulin (10 µg/mL), dexamethasone (1 nM), and IBMX (500 µM) for 2 days (days 0–2) and the medium was changed every 2 days. On day 4, overexpression and knockdown of miRNA-378 were done by transfecting the miRNA-378 mimic and/or inhibitor. On day 6, levels of miRNA-378 and mRNA levels of adiponectin, PPARγ_2_, PGC1α, ESRRG, PGC1β, ACSL1, and AGPAT6 were evaluated by qPCR. Expression levels normalized to those of β-actin were log-transformed. Individual values are shown with mean ± SE (n = 6). p values are shown for multigroup comparison by non-parametric Kruskal-Wallis test. *p<0.05 and †p<0.01 *vs*. day 6 for unpaired two group comparison by Wilcoxon-Mann-Whitney test.

Next, we evaluated the effects of overexpressing mimic or inhibitor of miRNA-378 on the expression of adiponectin, PPARγ_2_, PGC1α, ESRRG, PGC1β, ACSL1, and AGPAT6 (**Figure 3**). As shown, expression levels of miRNA-378 were effectively decreased by the inhibitor (p<0.05) and increased by the mimic (p<0.01). The expression of adiponectin was not significantly increased by miRNA-378 inhibitor but remarkably inhibited by mimic (p<0.01). Expression of ESRRG, alternatively targeted by miRNA-378, was also increased by miRNA-378 inhibitor and remarkably inhibited by mimic as previously reported by Eichner et al. [16]. The mimic and inhibitor of miRNA-378 had little or no effect on expression of PPARγ_2_, PGC1α, PGC1β, and AGPAT6, but the mimic decreased ACSL1 (p<0.05) significantly.

### Renilla-luciferase activity and site-directed mutagenesis

**Figure 4:**
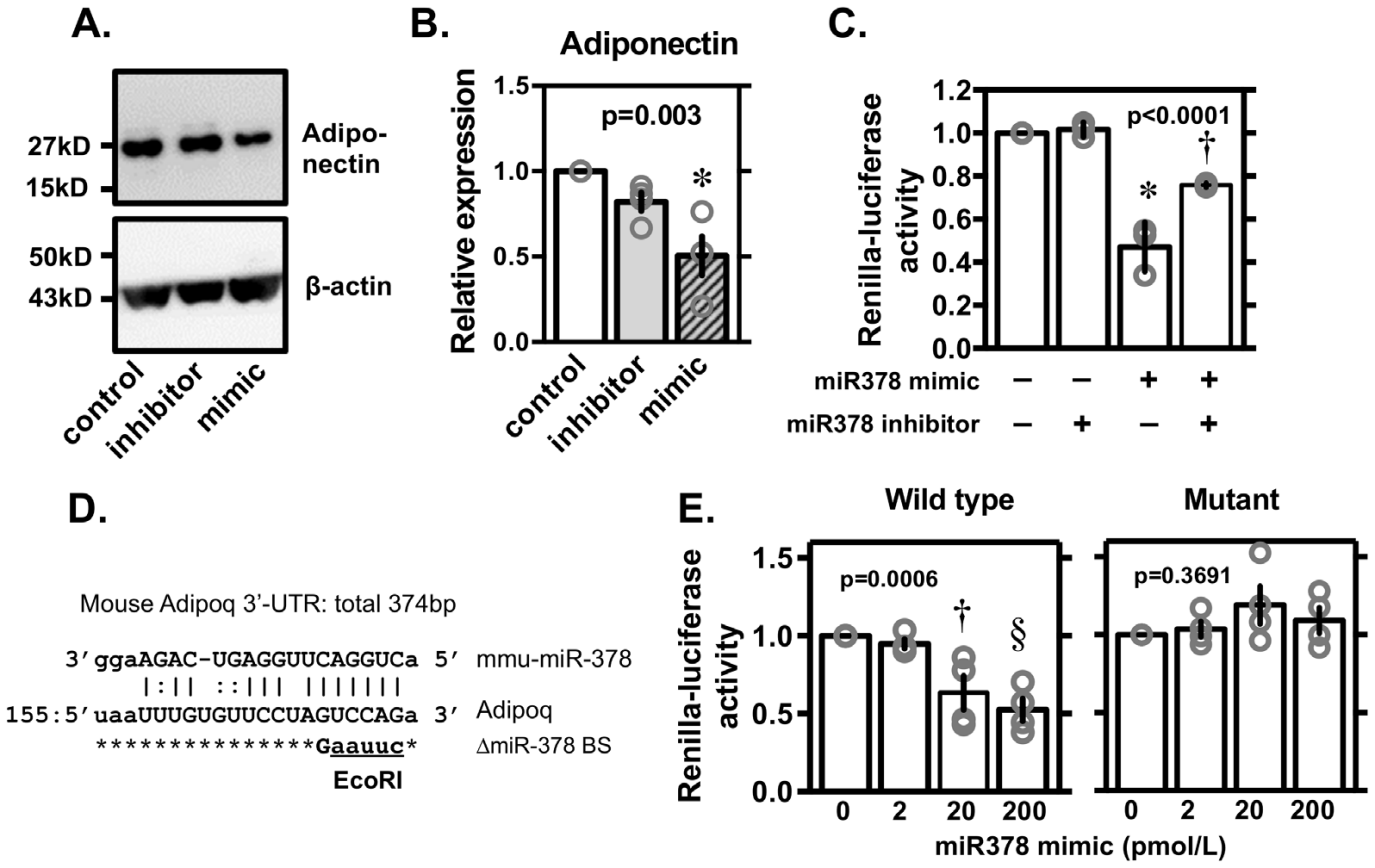
Representative blot (A), average expression levels of adiponectin protein (B) and Renilla-luciferase activity in HEK293T cells overexpressing the inhibitor or the mimic of miRNA-378 (C); Design of ΔmiR-378 binding site (BS) (D); Effects of overexpressing the mimic of miRNA-378R on the Renilla-luciferase activity in HEK293T cells with wild type or a deleted mutant of the miR-378 binding on 3′-UTR (E). Expression levels normalized to those of β-actin. Individual values are shown with mean ± SE (n = 4). p values are shown for multigroup comparison by non-parametric Kruskal-Wallis test. *p<0.05, †p<0.01 and §p<0.001 *vs*. controls or 0 for unpaired two group comparison by Wilcoxon-Mann-Whitney test.

We transfected the mimic and inhibitor of miR-378 into differentiating 3T3-L1 cells on day 4 and evaluated the levels of adiponectin protein on day 6 (**Figure 4A & 4B**), indicating that the mimic reduced adiponectin levels almost by half. To assess whether or not the putative miR-378 binding site was effectively utilized to regulate the expression of adiponectin, we transfected a renilla-luciferase-adiponectin-3'UTR sequence to HEK293T cells and further transfected the mimic and inhibitor of miRNA-378 (**Figure 4C**). Renilla-luciferase activity in HEK293T cells expressing the renilla-luciferase-adiponectin-3'UTR sequence was inhibited by overexpressing mimic of miRNA-378 and the decrease was partially reversed by adding the miRNA-378 inhibitor. Moreover, we confirmed the inhibitory effects of the mimic were cancelled in a deleted mutant of the miR-378 3′-UTR binding site (**Figure 4**D & **4**E).

### TNFα and miR378

**Figure 5:**
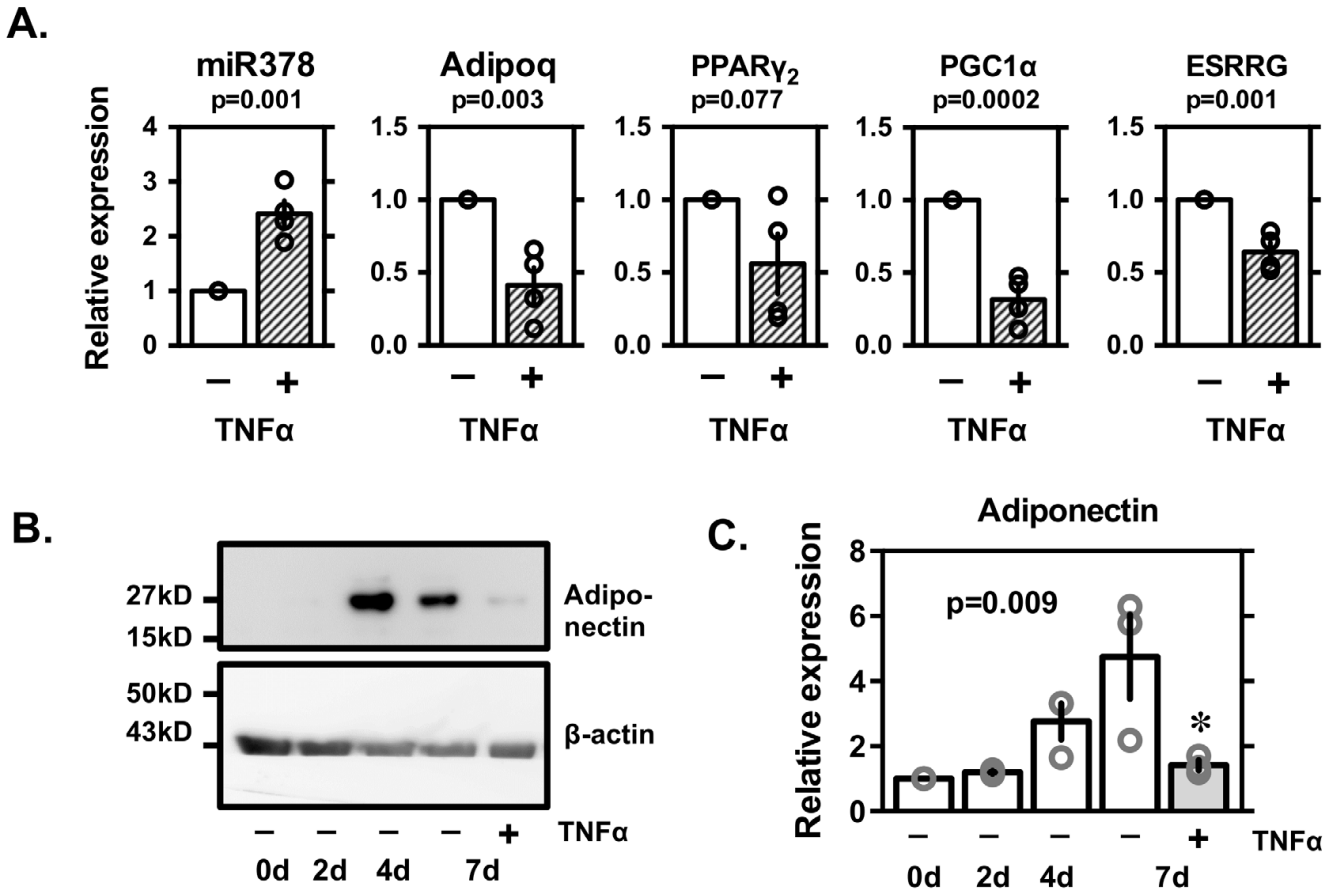
Level of miRNA 378 and mRNA levels of adiponectin, PPARγ_2_, PGC1α and ESRRG (A), representative blot (B) and average expression levels of adiponectin protein with or without TNFα in 3T3L1 adipocytes (C). 3T3L1 adipocytes were treated with human recombinant TNFα (10 µg/mL, R&D systems) on day 6 and miR-378 and mRNA levels were evaluated by qRT-PCR or Western blotting on day 7. Expression levels normalized to those of β-actin. Individual values are shown with mean ± SE (n = 4). p values are shown for intergroup comparison by non-parametric Kruskal-Wallis test. *p<0.05 *vs*. controls or 0 for unpaired two group comparison by Wilcoxon-Mann-Whitney test.

We analyzed that an inflammatory cytokine, TNFα, which is known as a negative regulator for adiponectin [6], may alter levels of miR-378 expression in 3T3-L1 adipocytes (**Figure 5**). Addition of TNFα led a significant upregulation of miR-378 (**Figure 5A**) and downregulation of adiponectin at mRNA and protein levels (**Figure 5A & 5B**). TNFα also decreased mRNA levels of PPARγ_2_, PGC1αand ESRRG (**Figure 5A**).

### miRNA-378 in C57BL/6 and ob/ob mice

**Figure 6:**
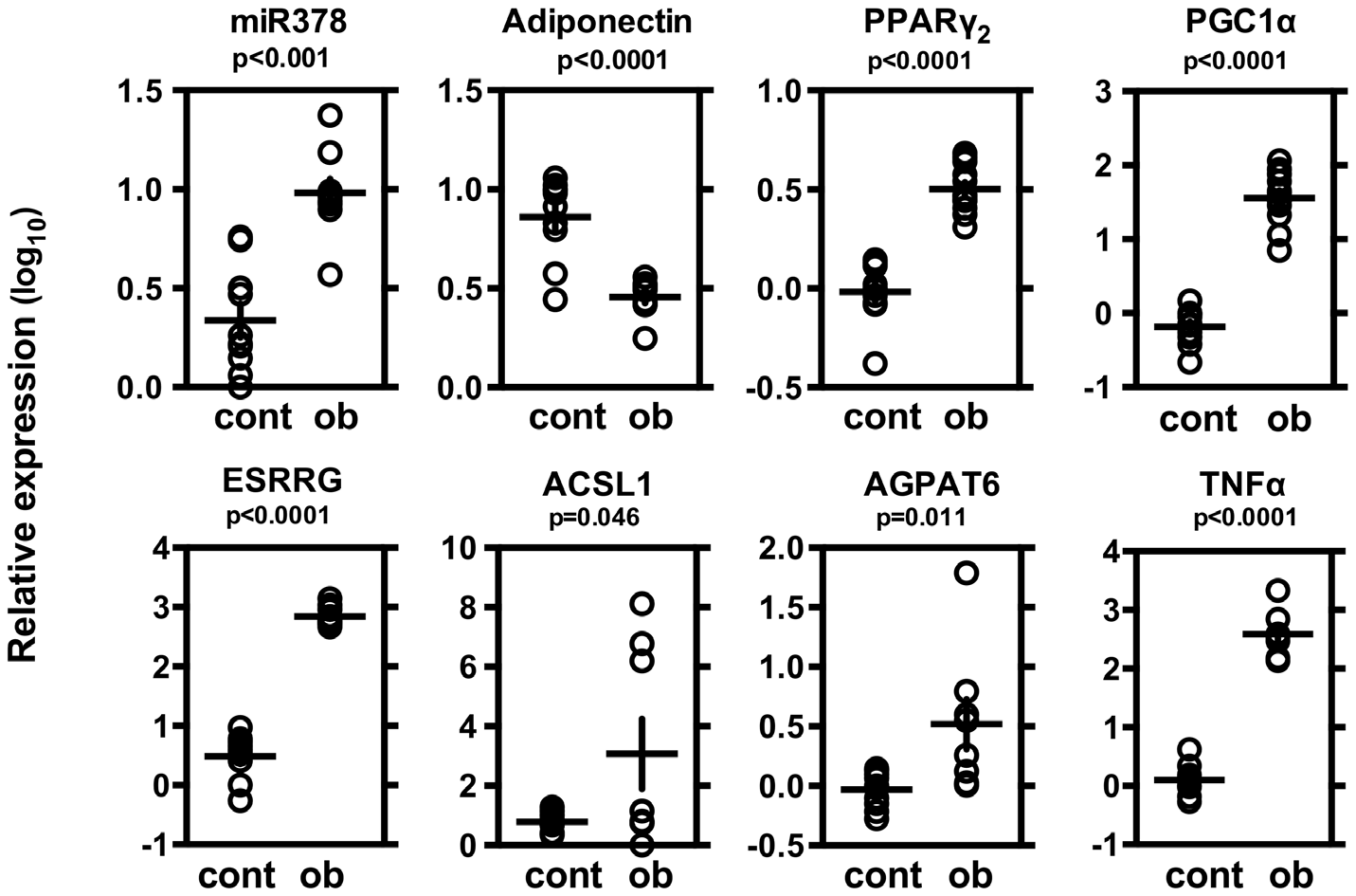
Levels of miRNA-378, adiponectin, PPARγ2, PGC1α, ESRRG, PGC1β, ACSL1, AGPAT6 and TNFα in the adipose tissue of C57BL/6 and ob/ob mice. From normal C57BL/6 (♂, n = 10) or diabetic ob/ob (♂, n = 10) mice, epididymal adipose tissues were excised and used for gene expression analyses. Expression levels normalized to those of β-actin were log-transformed. Individual values are shown with mean ± SE. p values were calculated by unpaired t-test.

**Figure 7:**
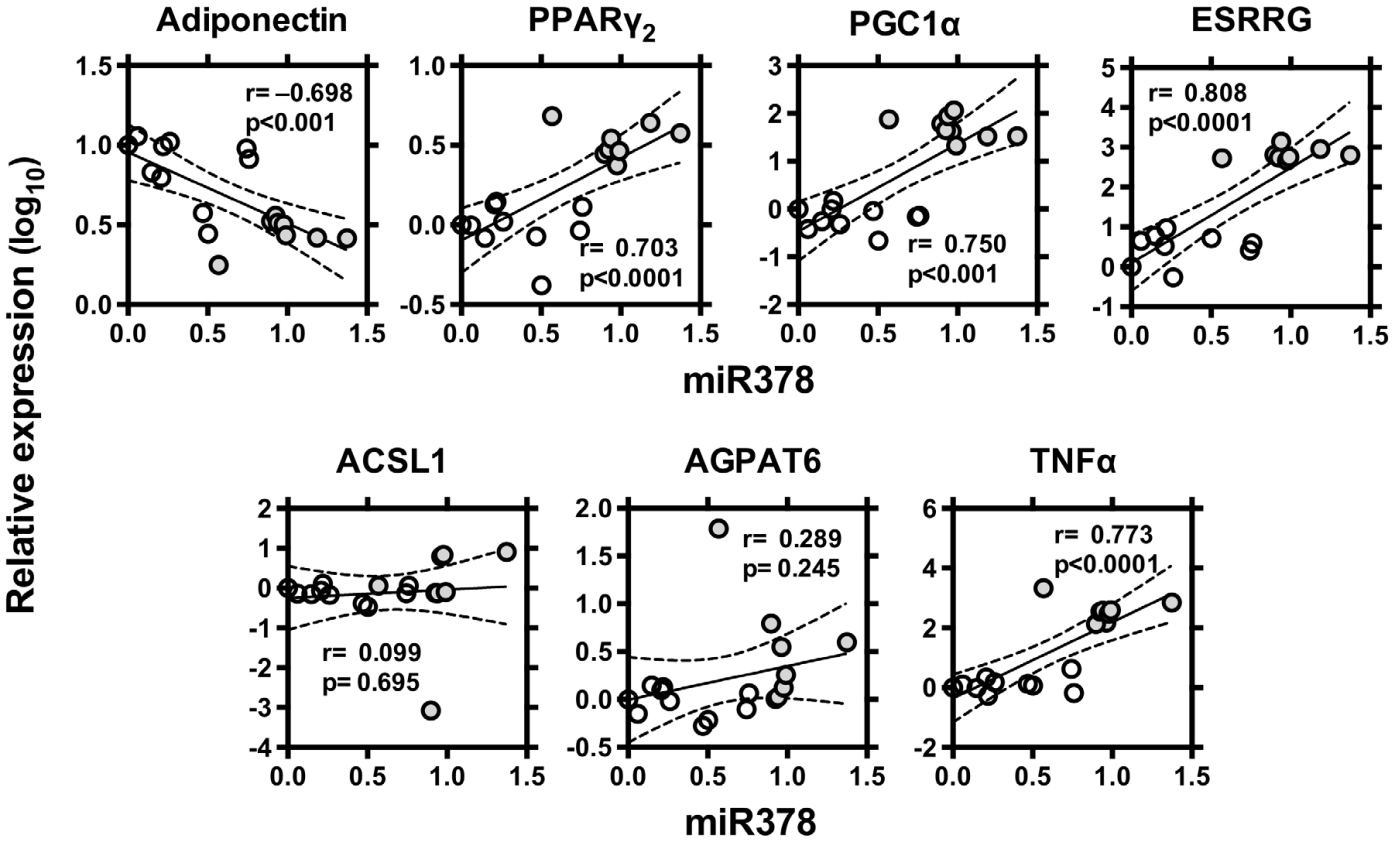
Correlation between level of miRNA-378 vs levels of adiponectin, PPARγ_2_, PGC1α, ESRRG, PGC1β, ACSL1, AGPAT6 and TNFα in the adipose tissue of C57BL/6 and ob/ob mice. From normal C57BL/6 (♂, n = 10) or diabetic ob/ob (♂, n = 10) mice, epididymal adipose tissues were excised and used for gene expression analyses. Expression levels normalized to those of β-actin were log-transformed. Linear regression analysis was made on a combined group of C57BL/6 (○) and ob/ob (•) mice and, and r and p values are shown.

**Figure 8:**
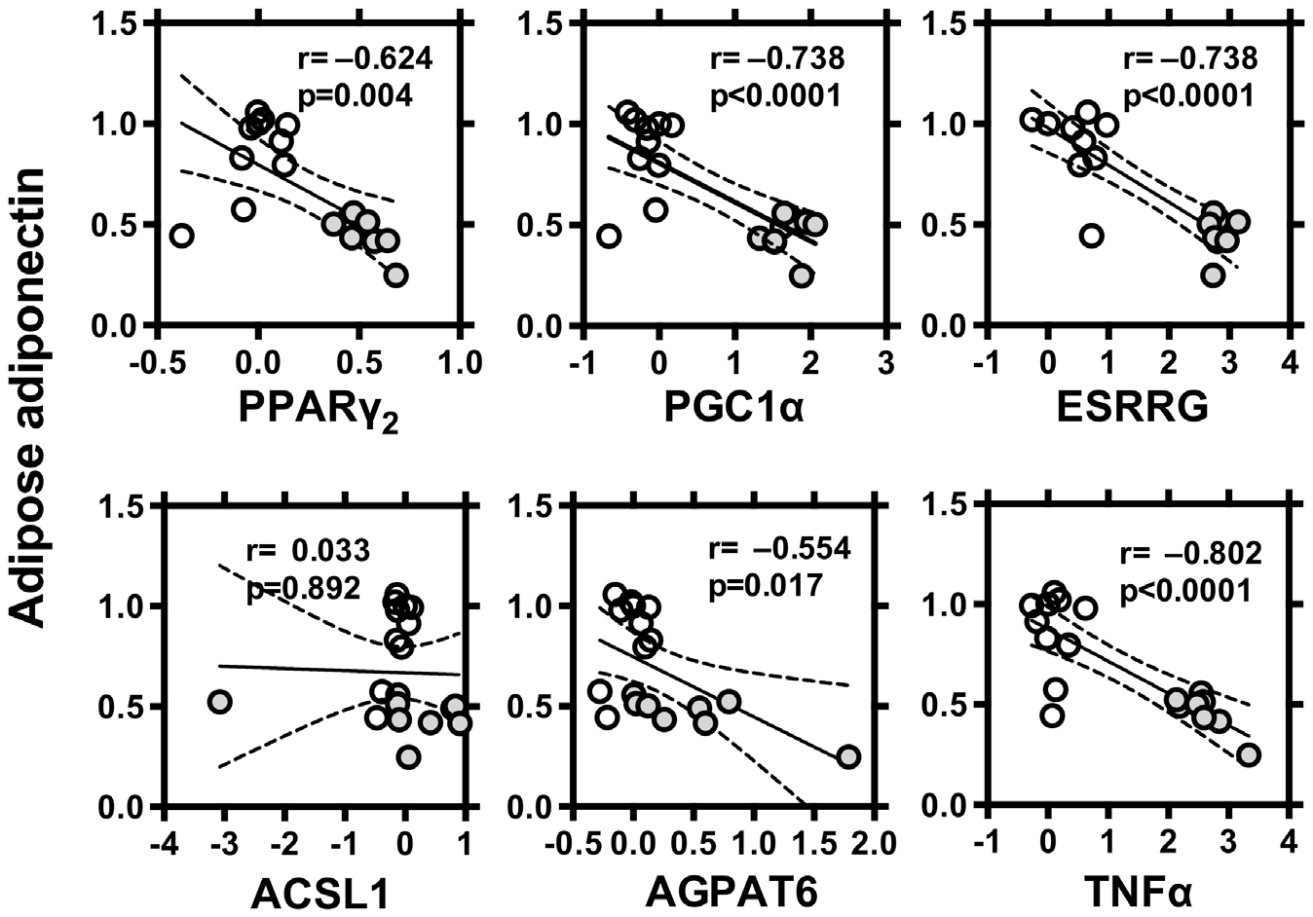
Correlation between level of adiponectin vs levels of PPARγ_2_, PGC1α, ESRRG, ACSL1, AGPAT6 and TNFα in the adipose tissue of C57BL/6 and ob/ob mice. From normal C57BL/6 (♂, n = 10) or diabetic ob/ob (♂, n = 10) mice, epididymal adipose tissues were excised and used for gene expression analyses. Expression levels normalized to those of β-actin were log-transformed. Linear regression analysis was made on a combined group of C57BL/6 (○) and ob/ob (•) mice and, and r and p values are shown.

Finally, we compared levels of miR-378, adiponectin and related molecules in white adipose tissue from normal C57BL/6 and ob/ob mice (**Figure 6** **–** **8**). Level of miR-378 was higher and level of adionectin was lower in ob/ob mice than those of C57BL/6. Levels of PPARγ_2_, PGC1α, ESRRG, ACSL1 and AGPAT6 were all higher in ob/ob mice (**Figure 6**). Level of miR378 was negatively correlated with mRNA levels of adiponectin. Level of miR378 was positively correlated with PPARγ_2_, PGC1α, ESRRG and TNFα, but not with levels of ACSL1 and AGPAT6 (**Figure 7**). Level of adiponectin was negatively correlated with mRNA levels of PPARγ_2_, PGC1α, ESRRG, AGPAT6 and TNFα but not with levels of ACSL1 (**Figure 8**).

## Discussion

In this study, we evaluated whether the miRNA-378 pathway is involved in regulation of adiponectin expression. **First**, we sought putative target sites for miRNAs in the 3'UTR of the adiponectin gene and found that miRNA-378 could target that region. **Second**, we found that intracellular levels of miRNA-378 were crucial in determining adiponectin expression, and that miRNA-378 could modulate adiponectin levels via the 3'UTR sequence-binding site.

Adiponectin gene (*Adipoq*) is known to be subject to several translational regulations. According to Liu et al., the expression of *Adipoq* is upregulated by multiple transcription factors such as PPARγ_2_, C/EBP, FoxO1, and SREBP [6]. On the other hand, *Adipoq* expression is downregulated by CREB and NFAT, and, more interestingly, by inflammatory cytokines such as IL-6, IL-18, and TNFα [6]. Recently, it was shown that the functional regulation or secretion of *Adipoq* requires proper polymerization and exocytosis [13], [17], [18]. Although pivotal transcription factors involved in *Adipoq* expression have been previously identified [6], the mechanisms for *Adipoq* regulation remain obscure. There are reports showing an association between miRNA and adiponectin [19], [20], but there are only few reports investigating the possibility that miRNAs could directly regulate *Adipoq* transcription [21]. We sought putative target sites for miRNAs in the 3'UTR of the adiponectin gene using TargetScan and PicTar and found that miRNA-378 could directly target the 3'UTR of adiponectin.

To confirm that miRNA-378 is crucial for the expression of adiponectin, and if so, how it regulates adiponectin expression, we performed the *in vitro* experiments described above. After 3T3-L1 adipocytes were transfected with the miRNA-378 inhibitor/mimic from day 4 through day 6, adiponectin expression was seen to be increased by the inhibitor and repressed by the mimic (**Figure 4**). Although the repressive influence of miRNA-378 on the adiponectin gene likely contributes to the lowering of adiponectin expression, the combined effects of the miRNA on multiple target genes might be involved [13], [15], [16], [22]. In 3T3-L1 cells, both adiponectin and miR-378 are increased during differentiation into adipocytes, suggesting that miR-378 is not a suppressor of adiponectin mRNA expression during normal differentiation. On the other hand, the expression levels of mirR-378 and adiponectin were negatively well correlated in 3T3-L1 adipocytes after treated with TNFα. Such negative correlation was also observed in comparison between adipose tissues among normal C57BL/6 and diabetic ob/ob mice. It might be suggested that miR-378 regulates adiponectin expression in a pathological condition. From the current study, it is not clear why miR-378 is upregulated by TNFα. It is interesting that upregulation of miR-378 and downregulation of adiponectin by TNFα mimic the phenotype of diabetic ob/on mice [23]. Relevance of miR-378 in the pathogenesis of obesity and diabetes mellitus should be evaluated in the future study. Especially, genetic variation of relevant 3′UTR sequence in human subjects with hyper or hypoadiponectinemia should be explored.

Gerin et al. reported that overexpression of miR-378 leads to increased lipid droplet size, lipogenesis, and expression of PPARγ_2_ and Glut4 [13]. They further described that miR-378 induces transactivation of C/EBP, indicating that miR-378 can act through a mechanism independent of the classical miRNA machinery. Taken above, miR-378 might regulate adiponectin expression through a C/EBP binding site of the adiponectin promoter [13]. Carrer et al. showed that mice genetically lacking miRNA-378 are resistant to obesity induced by a high-fat diet, and exhibit enhanced mitochondrial fatty acid oxidation [15]. miRNA-378-mediated repression of carnitine O-acetyltransferase (CRAT) [15], a mitochondrial enzyme involved in fatty acid oxidation, and MED13, a component of the mediator complex that controls nuclear hormone receptor activity [15], and insulin-like growth factor 1 receptor [22], might at least partly contribute, to the elevated oxidative capacity. Moreover, miRNA-378 has been shown to target mRNAs encoding ESRRG and GA-binding protein-α, which associates with PGC-1β to control oxidative metabolism [16]. In our C57BL/6 and ob/ob study, level of miR-378 was positively correlated with lipogenic molecules such as PPARγ_2_, PGC1α, ESRRG and AGPAT and also with a negative regulator for adiponectin, TNFα. Taken together, miR-378 could play a role in pathophysiological conditions via the regulation of adiponectin expression discussed below.

To determine whether adiponectin expression was directly regulated by miRNA-378, we measured the effects of the inhibitor or mimic of miRNA-378 on renilla-luciferase activity in HEK293T cells expressing a renilla-luciferase-adiponectin-3'UTR sequence. Renilla-luciferase activity was inhibited by overexpressing the mimic of miRNA-378, and the decrease was partially reversed by adding the miRNA-378 inhibitor. Since the inhibitory effects of the mimic were cancelled in a deleted mutant of the miR-378 3′-UTR binding site (**Figure 4**D & **4**E), suggesting that the regulation of adiponectin expression by miR378 can occur via the adiponectin-3'UTR sequence.

Our study has several limitations. First, the suitability of the 3T3-L1 and HEK293T cell lines needs to be taken into account. The 3T3-L1 adipocyte model has limitations compared with freshly prepared adipose tissue cells [23]: (i) generation of 3T3-L1 adipocytes from preadipocytes requires at least 2 weeks; (ii) if 3T3-L1 cells become over-propagated or are passaged extensively, they no longer differentiate robustly into adipocytes; (iii) it is not possible to efficiently detect mRNAs, miRNAs, and proteins expressed from transiently transfected DNA in 3T3-L1; and (iv) because the 3T3-L1 cell line has clone-specific traits, it fails to recapitulate the primary cells. HEK293T cells, used because of the low transfection efficiency of 3T3-L1 adipocytes, differ from adipocytes in transfection activity and gene response. Second, the results from a model overexpressing a miRNA-378 mimic/inhibitor may not be relevant to natural physiological systems.

In conclusion, expression of adiponectin can be modulated by miR-378 via the 3'UTR miRNA-378 binding site. All these above researches on miR-378 and metabolic events were demonstrated in rodent models so far, in future our findings warrant further investigations into the role of miRNA-378 and other miRNAs possibly affecting the adiponectin expression in human subjects, which is linked to metabolic and cardiovascular abnormalities associated with obesity and insulin resistance.

## Supporting Information

Table S1 **Primers used for this study.**
(DOCX)

## References

[1] Matsuzawa Y (2006) Therapy Insight: adipocytokines in metabolic syndrome and related cardiovascular disease. Nat Clin Pract Cardiovasc Med 3: 35–42. 16391616 10.1038/ncpcardio0380

[2] Kadowaki T, Yamauchi T, Waki H, Iwabu M, Okada-Iwabu M, et al (2011) Adiponectin, adiponectin receptors, and epigenetic regulation of adipogenesis. Cold Spring Harb Symp Quant Biol 76: 257–265. 22492282 10.1101/sqb.2012.76.010587

[3] Dusserre E, Moulin P, Vidal H (2000) Differences in mRNA expression of the proteins secreted by the adipocytes in human subcutaneous and visceral adipose tissues. Biochim Biophys Acta 1500: 88–96. 10564721 10.1016/s0925-4439(99)00091-5

[4] Perrini S, Laviola L, Cignarelli A, Melchiorre M, De Stefano F, et al (2008) Fat depot-related differences in gene expression, adiponectin secretion, and insulin action and signalling in human adipocytes differentiated in vitro from precursor stromal cells. Diabetologia 51: 155–164. 17960360 10.1007/s00125-007-0841-7

[5] Hiuge-Shimizu A, Maeda N, Hirata A, Nakatsuji H, Nakamura K, et al (2011) Dynamic changes of adiponectin and S100A8 levels by the selective peroxisome proliferator-activated receptor-gamma agonist rivoglitazone. Arterioscler Thromb Vasc Biol 31: 792–799. 21233451 10.1161/ATVBAHA.110.221747

[6] Liu M, Liu F (2010) Transcriptional and post-translational regulation of adiponectin. Biochem J 425: 41–52. 10.1042/BJ20091045 20001961

[7] Shimabukuro M, Higa N, Asahi T, Oshiro Y, Takasu N, et al (2003) Hypoadiponectinemia is closely linked to endothelial dysfunction in man. J Clin Endocrinol Metab 88: 3236–3240. 12843170 10.1210/jc.2002-021883

[8] Ouchi N, Parker JL, Lugus JJ, Walsh K (2011) Adipokines in inflammation and metabolic disease. Nat Rev Immunol 11: 85–97. 21252989 10.1038/nri2921 PMC3518031

[9] Guo H, Ingolia NT, Weissman JS, Bartel DP (2010) Mammalian microRNAs predominantly act to decrease target mRNA levels. Nature 466: 835–840. 20703300 10.1038/nature09267 PMC2990499

[10] Filipowicz W, Bhattacharyya SN, Sonenberg N (2008) Mechanisms of post-transcriptional regulation by microRNAs: are the answers in sight? Nat Rev Genet 9: 102–114. 18197166 10.1038/nrg2290

[11] Xie H, Lim B, Lodish HF (2009) MicroRNAs induced during adipogenesis that accelerate fat cell development are downregulated in obesity. Diabetes 58: 1050–1057. 19188425 10.2337/db08-1299 PMC2671055

[12] Rantalainen M, Herrera BM, Nicholson G, Bowden R, Wills QF, et al (2011) MicroRNA expression in abdominal and gluteal adipose tissue is associated with mRNA expression levels and partly genetically driven. PLoS One 6: e27338. 22102887 10.1371/journal.pone.0027338 PMC3216936

[13] Gerin I, Bommer GT, McCoin CS, Sousa KM, Krishnan V, et al (2010) Roles for miRNA-378/378* in adipocyte gene expression and lipogenesis. Am J Physiol Endocrinol Metab 299: E198–206. 20484008 10.1152/ajpendo.00179.2010 PMC2928515

[14] Spiegelman BM (2013) Banting Lecture 2012: Regulation of adipogenesis: toward new therapeutics for metabolic disease. Diabetes 62: 1774–1782. 23704518 10.2337/db12-1665 PMC3661621

[15] Carrer M, Liu N, Grueter CE, Williams AH, Frisard MI, et al (2012) Control of mitochondrial metabolism and systemic energy homeostasis by microRNAs 378 and 378*. Proc Natl Acad Sci U S A 109: 15330–15335. 22949648 10.1073/pnas.1207605109 PMC3458360

[16] Eichner LJ, Perry MC, Dufour CR, Bertos N, Park M, et al (2010) miR-378(*) mediates metabolic shift in breast cancer cells via the PGC-1beta/ERRgamma transcriptional pathway. Cell Metab 12: 352–361. 20889127 10.1016/j.cmet.2010.09.002

[17] Phillips SA, Kung JT (2010) Mechanisms of adiponectin regulation and use as a pharmacological target. Curr Opin Pharmacol 10: 676–683. 20810317 10.1016/j.coph.2010.08.002

[18] Engeli S, Feldpausch M, Gorzelniak K, Hartwig F, Heintze U, et al (2003) Association between adiponectin and mediators of inflammation in obese women. Diabetes 52: 942–947. 12663465 10.2337/diabetes.52.4.942

[19] Santovito D, De Nardis V, Marcantonio P, Mandolini C, Paganelli C, et al.. (2014) Plasma exosome microRNA profiling unravels a new potential modulator of adiponectin pathway in Diabetes: effect of glycemic control. J Clin Endocrinol Metab: jc20133843. 10.1210/jc.2013-3843 24937531

[20] Li H, Chen X, Guan L, Qi Q, Shu G, et al (2013) MiRNA-181a regulates adipogenesis by targeting tumor necrosis factor-α (TNF-α) in the porcine model. PLoS One 8: e71568. 24098322 10.1371/journal.pone.0071568 PMC3787936

[21] Kang M, Yan LM, Zhang WY, Li YM, Tang AZ, et al (2013) Role of microRNA-21 in regulating 3T3-L1 adipocyte differentiation and adiponectin expression. Mol Biol Rep 40: 5027–5034. 23793828 10.1007/s11033-013-2603-6

[22] Knezevic I, Patel A, Sundaresan NR, Gupta MP, Solaro RJ, et al (2012) A novel cardiomyocyte-enriched microRNA, miR-378, targets insulin-like growth factor 1 receptor: implications in postnatal cardiac remodeling and cell survival. J Biol Chem 287: 12913–12926. 22367207 10.1074/jbc.M111.331751 PMC3339988

[23] Müller G (2002) Insulin target cells/tissues of rats; HG V, editor. Heidelberg: Springer-Verlag Berlin.

