# Supplementary material for: MicroRNA-378 Regulates Adiponectin Expression in Adipose Tissue: A New Plausible Mechanism
Source: PLoS One. 2014 Nov 7;9(11):e111537. doi: 10.1371/journal.pone.0111537 (PMC4224402; doi:10.1371/journal.pone.0111537)
Supplement: Table S1 — Primers used for this study. (DOCX) [file pone.0111537.s001.docx]

**Supplemental Table S1.** Primers used for this study.

| Genes | sequence |
| --- | --- |
| Adipoq | Forward GATGGCAGAGATGGCACTCC |
|  | Reverse: CTTGCCAGTGCTGCCGTCAT |
| PPARγ_2_ | Forward:CACCAGTGTGAATTACAGCAAATC |
|  | Reverse: AAGCGATTCCTTCACTGATACAC |
| PGC1α | Forward: GCCAAACCAACAACTTTATCTCTTC |
|  | Reverse: CACACTTAAGGTGCGTTCAATAGTC |
| PGC1β | Forward CCAAGACCAGCAGCTCCTACGG |
|  | Reverse: AGTTGGGTCGCTTTGTGACAAG |
| ESRRG | Forward CTGGTAAAGAAATACAAGAGCATGAAGC |
|  | Reverse: CAGCATCTTGCCAGCTCGACGAGGGTCT |
| ACSL1 | Forward TGGGGTGGAAATCATCAGCC |
|  | Reverse: CACAGCATTACACACTGTACAACGG |
| AGPAT6 | Forward GGCAGAGGAGCTGGAGTC |
|  | Reverse: TGTTGTGGTACGTAATGATGG |
| TNFα | Forward: ACCCTCACACTCAGATCATCTTC |
|  | Reverse: TGGTGGTTTGCTACGACGT |
